# Health Behavior-Related Correlates of Physical and Mental Health Among Potential Conscripts

**DOI:** 10.3390/nu17203214

**Published:** 2025-10-14

**Authors:** Brigita Mieziene, Kristina Motiejunaite, Arunas Emeljanovas

**Affiliations:** Department of Physical and Social Education, Lithuanian Sports University, Sporto Str. 6, 44221 Kaunas, Lithuania; kristina.motiejunaite@lsu.lt (K.M.); arunas.emeljanovas@lsu.lt (A.E.)

**Keywords:** conscripts’ health, physical fitness, health behavior

## Abstract

**Background**: The nation’s defense relies on a cadre of young individuals with strong physical and mental health. The study aimed to identify health behavior-related correlates of physical and mental health in future conscripts. **Methods**: This cross-sectional study comprised 676 male school students with an average age of 18.48 (1.15) years. The measures of weekly physical activity, nutrition (Kidmed questionnaire), psychological well-being (WHO-5 Well-Being Index), psychological distress (Kessler scale), cardiorespiratory fitness (20 m Shuttle Run test), weight, and height for calculation of body mass index were taken. **Results**: Among potential conscripts (17–19-year-old males), 22% have poor cardiorespiratory fitness. More than a quarter of young males have poor psychological well-being. Better cardiorespiratory fitness is related to more recommendations-adherent nutrition (Std β = 0.133 [0.03–0.14], *p* < 0.05). Engagement in sports is related to better cardiorespiratory fitness (Std β = 0.202 [0.10–0.30], *p* < 0.05). Higher psychological distress is associated with more time spent passively (Std β = 0.145 [0.12–0.34], *p* < 0.01); better psychological well-being is associated with more adherent nutrition (Std β = 0.172 [0.14–0.34], *p* < 0.0001), more hours accumulated in moderate-to-vigorous physical activity (Std β = 0.150 [0.30–1.10], *p* < 0.01), and less time spent passively (Std β = −0.131 [−0.34–−0.11]). **Conclusions**: Potential conscripts’ physical fitness and mental health are related to better health behaviors. Behavior change practices and policies applied at school could benefit potential conscripts and youth in general in terms of their physical and mental health.

## 1. Introduction

In Lithuania, a small country in Europe with a resident population of 2.9 mln. [1], young people aged 18 to 26 are conscripted into military service for nine months in accordance with the Law on Mandatory Military Service of the Republic of Lithuania [2]. The nation’s defense relies on a cadre of young individuals with strong physical and mental health [3]. Mental and physical well-being are key criteria for evaluating young men’s eligibility for military service, as they are closely linked to the ability to perform military duties. A previous pre-COVID-19 study found that nearly one in ten conscripts experienced high psychological distress [3]. Another post-COVID-19 study conducted in Lithuania showed even more concerning numbers, as 21.01% of conscripts were at high risk for suicide [4]. The rates of poor psychological well-being, along with the risk for depression after the COVID-19 lockdown, reached up to 42% among middle and high school students [5]. Along with mental health, physical fitness, particularly cardiorespiratory fitness, has deteriorated each decade since 1992 [6]. Similar secular trends have been observed in other European countries, where declines in cardiorespiratory fitness and increases in obesity among youth raise concerns for both public health and military readiness (Tomkinson et al., 2019) [7].

Studies in other countries examining the health of conscripts have also revealed alarming results, similar to those in Lithuania. For example, a study in Finland found that 9.4% of conscripts were discharged early for medical reasons, primarily due to musculoskeletal, mental, and behavioral disorders [8]. Studies have also found that conscripts with insufficient muscle strength, low cardiorespiratory fitness, and excessive body weight are more susceptible to developing musculoskeletal injuries and disorders [9]. These findings highlight the concerning state of physical and mental health among contemporary young people eligible for military service. Moreover, subjective perceptions of physical and mental health have deteriorated as a result of the recent global crisis. The HBSC study in Germany revealed a significant decline in adolescents’ self-reported health and life satisfaction when comparing data from before and after the COVID-19 pandemic [10].

Alongside the declining mental and physical health of conscripts and male youth in general, research also shows a decrease in health-promoting behaviors among young individuals, which adversely affects their overall well-being. A comprehensive investigation into the lifestyle factors of Estonian conscripts has elucidated the pivotal role of pre-enlistment lifestyle factors in determining soldiers’ physical fitness levels. The study’s findings indicate that health-related behaviors have a significant impact on performance in military fitness assessments [11]. Moreover, studies suggest that, in addition to assessing health behaviors, stress management skills should also be incorporated into the pre-screening questionnaire [12]. Evaluating these skills can help identify individuals who may be at risk of heightened stress or poor coping mechanisms, which can affect both their mental well-being and physical performance during military service. Recent evidence confirms that maladaptive coping strategies are significant risk factors for stress, burnout, depression, and PTSD among military and law enforcement personnel [13]. Including stress management assessment could improve early identification of support needs and enhance overall readiness and resilience among conscripts.

A pre-COVID-19 study in Lithuania found that among all conscripts, just over half (55.3%) were physically active, 50.2% had low adherence to a healthy diet, one in ten were heavy drinkers, and more than half (62.3%) were smokers [3]. Alcohol consumption levels were similar across the general male population, conscripts, and regular soldiers; however, it was a significant predictor of suicide risk in all three groups [4]. Additionally, a higher body mass index is not only a significant indicator of physical health [14,15,16] but is also strongly associated with lower levels of physical activity, increased disordered and comfort eating, and higher alcohol consumption [17].

It is obvious that the prevalence of detrimental behaviors undermines individual psychological well-being and physical health and carries profound implications for the nation’s defense capabilities. Physical inactivity, poor nutrition, substance use, and psychological distress can all contribute to reduced fitness levels and increased health risks, potentially compromising the readiness and effectiveness of military personnel [3,7]. It is critically important to investigate the impact of behavioral factors on health indicators in the post-COVID-19 pandemic context because the relationships observed after the pandemic may differ considerably from those identified in pre-pandemic research. The pandemic has introduced unprecedented social, psychological, and health-related challenges that could alter the dynamics and interactions of these factors. Therefore, studying these impacts post-pandemic will provide more accurate and relevant insights into how these variables function under changed global circumstances, contributing to more effective public health strategies and interventions tailored to the current realities. This research is essential for understanding the long-term effects of the pandemic on mental and physical health as well as behavioral patterns.

This study aims to investigate the health behavior correlates of physical and mental health among potential conscripts. What behavioral factors, and to what extent, are important for physical and mental health among young males in the post-COVID-19 context? By examining the associations between nutrition, physical activity, and indicators of both physical and mental health, we seek to clarify the importance of these factors within this population. Understanding these relationships is crucial for developing targeted interventions that enhance the health status of potential conscripts, ultimately improving military preparedness and broader public health outcomes.

Our research builds on previous findings indicating that physically active individuals are more likely to be enlisted for military service and tend to maintain better physical fitness throughout their service period [3,11]. Moreover, we explore the relationship between health behavior and mental health, a factor increasingly recognized as crucial for adapting to the demands of military service [18].

By addressing these critical issues, this study contributes valuable insights to the fields of public health, military medicine, and exercise science, ultimately informing evidence-based strategies to promote health and fitness among young adults eligible for conscription.

## 2. Materials and Methods

### 2.1. Participants

This cross-sectional study consisted of 676 male school students, with an average age of 18.48 (1.15). This study is part of a bigger research project and included only school students who, along with their objective measures, had their subjective measures taken as well.

### 2.2. Study Design and Procedure

Study participants were selected using nested sampling. At least two schools were randomly selected in each of the ten counties of Lithuania, either the county center or its region. In total, 20 schools were included. In each school, grades 11 and 12 were selected, and all students in the chosen grades were measured following the EUROFIT tests’ instructions and asked to fill out the online study questionnaires (for about 30 min to fill in) following the provided link, unless their parents had not signed parental consent. The number of students in each school varied from 65 to 75.

Permission to conduct the study was obtained from the Committee of Bioethics at Lithuanian Sports University (permission no. 27720). All participants included in the study had parental consent and willingly agreed to participate. The study was performed following the Helsinki ethical guidelines.

### 2.3. Instruments

#### 2.3.1. Physical Activity

Moderate-to-vigorous physical activity (MVPA) was measured by the question, “On average, how much time per week do you spend exercising or doing sports that make you sweat and breathe faster?”. The number of hours and minutes each participant spent in MVPA was allocated either to group (1) of those who do not and (2) those who do meet health-related physical activity requirements by the World Health Organization. The threshold for 17-year-olds was 7 h per week, and for 18-year-olds and older students it was <2.5 h/week [19].

#### 2.3.2. Time Spent Passively

Time spent passively was evaluated by the question “Please identify the average time per day you spent sitting while at school, at home, while doing coursework, and during leisure time”. The number of hours identified was used as a continuous variable in calculations.

#### 2.3.3. Sports Engagement

Students were asked if they participated in any kind of organized sports where they are physically active at a moderate-to-vigorous level after school. The question was binary and contained answers “No” and “Yes”.

#### 2.3.4. Nutrition

An updated Kidmed questionnaire [20] was used to measure dietary habits among study participants. The questionnaire contains 16 statements with binarized answers (0)—“No” and (1)—“Yes”. Participants were asked to rate their dietary habits over seven days. The total score was the sum of points of each “Yes” in items 1–5, 7–11, 13, and 15, while each “Yes” in items 6, 12, 14, and 16 reduced the total score by 1 point. Three groups were derived based on their total score: ≤3 points—inadequate diet; 4–7 points—an average diet; and ≥8 points—a good diet. The original version of the Kidmed questionnaire was previously used in several studies among Lithuanian schoolchildren [21,22].

#### 2.3.5. Mental Health

Psychological well-being was assessed using The World Health Organisation-Five Well-Being Index (WHO-5) 5-item questionnaire [23]. The questionnaire identified the frequency within the previous two weeks of being active, vigorous, rested, relaxed, having interests, and being in good spirits. Answers ranged on the Likert scale from 0—“none of the time” to 5—“all of the time”. The summed total score was multiplied by 4 as proposed to have a range of scores from 0 to 100. The higher score indicated better psychological well-being. The instrument has been found to have adequate validity in screening for depression and in measuring outcomes in clinical trials [23]. The internal consistency in the current study was Cronbach’s α 0.871, indicating good internal consistency.

Psychological distress has been evaluated using Kessler’s six-item scale [24]. Participants rated their nervousness, hopelessness, anxiety, restlessness or fidgety feelings, worthlessness, and depression on a scale from 0 (none of the time) to 4 (all the time). The total score was obtained by summing the scores for each item. A higher score indicates a higher level of psychological distress. The scale has a good internal consistency (Cronbach α = 0.888).

#### 2.3.6. Physical Health

Body mass index (BMI) was calculated by dividing body mass (kg) by height squared (m^2^), which was measured using digital weighing scales and stadiometers, respectively. BMI groups were allocated according to Cole and colleagues’ (2000) reference norms for age and male gender [25].

Cardiorespiratory fitness was assessed by a 20 m Shuttle Run test from the EUROFIT test battery [26]. Participants’ cardiorespiratory fitness was indicated by the number of stages covered before “falling out” (end of the test) (1 stage is equal to 1 min; 10 stages—10 min). The higher the score, the greater the cardiorespiratory endurance. Thresholds for the “Risk zone”, “Needs improvement”, and “Healthy fitness” zones were applied according to the “Order on Approval of the Procedure for Determining the Physical Fitness of Students Studying According to Primary, Basic, and Secondary Education Programs” (https://sam.lrv.lt/uploads/sam/documents/files/%C4%AEsakymas.pdf, accessed on 24 August 2025), which provides reference thresholds for Lithuanian school students.

### 2.4. Statistical Analysis

SPSS 28.0 (SPSS Inc., Chicago, IL, USA) and MPLUS 8.4 software were employed to analyze data. Descriptive statistics in terms of means (SD) and frequencies were used. A series of linear regressions were performed to identify relationships between behavioral factors and physical and mental health indicators. Logistic regression was performed for the binary BMI outcome. Statistical significance was set at a *p*-value of less than 0.05.

## 3. Results

Results presented in Table 1 reveal that among potential conscripts, i.e., a population of 18–19-year-old males, 22% have poor cardiorespiratory fitness, and one-third belong to the group of good cardiorespiratory fitness. The rest are in the “needs improvement” zone. Descriptive statistics in Table 1 also show that almost one out of five potential conscripts is either overweight or obese, and 7 percent are underweight. Regarding mental health, more than a quarter of young males have either poor psychological well-being and are at risk of depression or have high psychological distress. Looking at the health behaviors, almost a quarter of potential conscripts lack proper nutrition, more than one-third comply with nutrition recommendations, and the rest have average nutrition. Even though more than two-thirds of young males are engaged in sports after school, only about one-third accumulate a sufficient amount of moderate-to-vigorous physical activity by the WHO’s physical activity recommendations for adults from 18 to 65 years old (Table 1).

The results presented in Table 2 disclose that better cardiorespiratory fitness is related to more recommendations-adherent nutrition, but nutrition is also related to being overweight or obese. Engagement in sports, but not moderate-to-vigorous physical activity, and not time spent passively, are related to better cardiorespiratory fitness. Higher psychological distress is related to more time spent passively, but not to physical activity or engagement in sports, or to nutrition. However, better psychological well-being is associated with more adherence to recommendations, more hours spent engaging in moderate-to-vigorous physical activity, and less time spent passively. Behavioral factors better explain psychological well-being, accounting for 11% of its variance, than other health outcomes. Meanwhile, the proportion of variance explained for other outcome variables ranges from 3.3% to 5.4%.

The correlations between outcome variables were examined. Both physical health indicators and mental health indicators were found to be interrelated. Associations within each health domain were stronger than those between domains. Higher cardiorespiratory fitness was associated with lower psychological distress, while higher BMI was associated with greater psychological distress. No significant associations were observed between physical health indicators and psychological well-being (Table 3).

## 4. Discussion

This study aimed to identify health behavior-related predictors of physical and mental health among future conscripts. Recent changes to the law on Mandatory Military Service in Lithuania designate all male high school students as potential candidates for military service. Their health readiness will be assessed during their final year of high school, beginning at age 18. This study provides a baseline assessment of the physical and mental health of future conscripts prior to the implementation of the new law. It also highlights health predictors that could be addressed early in the school setting, as well as the public health challenges that need to be overcome.

The study confirmed the relationships between health behaviors and indicators of physical and mental health. In particular, adherence to the Mediterranean diet was associated with improved cardiorespiratory fitness and higher levels of psychological well-being. Although this study did not investigate causal relationships, other cross-sectional research has reported that higher diet quality is associated with better cardiorespiratory fitness. However, those studies were conducted in middle-aged community-dwelling populations, where dietary habits have had more time to influence physical fitness outcomes [27]. The *Lancet* published a study emphasizing the critical importance of nutrition during adolescence for the development of physiological systems, suggesting that adolescence represents a nutrition-sensitive window crucial for promoting healthy growth [28]. Moreover, nutritional habits combined with cardiorespiratory fitness account for 20% of the variance in body mass index among children in grades one through five [29]. Earlier findings specify that higher cardiorespiratory fitness is associated with greater consumption of dairy products and bread/cereals, along with lower intake of sweetened beverages. [30].

The relationship between nutrition and mental health is well-established in the scientific literature. The importance of specific micro- and macronutrients, within the context of a well-balanced and varied diet combined with a healthy lifestyle, has been recognized as essential for supporting normal brain function and overall well-being [31]. Another study found that the adoption of a polyphenol-rich diet could potentially lower depressive symptoms and improve general mental health status, as well as physical health [32]. Polyphenols help combat oxidative stress, which is linked to various mental health disorders [33]. As far as the Mediterranean diet is rich in polyphenols, this may explain its relationship with greater psychological well-being in the current study. Health-related behaviors were associated with greater psychological well-being in other studies [34].

However, the study also found that better adherence to the Mediterranean diet was associated with overweight or obesity among school students eligible for military service. One possible explanation is that individuals who generally consume larger quantities of food also tend to consume more of the healthy items, leading to higher scores on the diet adherence scale. The Kidmed instrument assesses whether participants meet minimum thresholds for healthy food items and stay below maximum thresholds for unhealthy items, but it does not quantify the exact amounts consumed.

When examining the distribution of BMI groups for each item, overweight or obese students reported eating fresh or boiled vegetables more than once per day (49.8% vs. 39.5% for non-overweight) and legumes more frequently (48.8% vs. 38.2%). They also consumed fast food less often (18.6% vs. 27.1%). Despite these differences, the lack of precise measurement of food quantity means the calorie intake for each BMI group remains unknown. Therefore, future research should adopt a more detailed nutritional assessment to better understand the relationship between healthy eating patterns and overweight status.

Moderate-to-vigorous physical activity (MVPA) in this study was not associated with cardiorespiratory fitness, body mass index, or psychological distress, but it was positively associated with psychological well-being. Specifically, higher levels of MVPA corresponded to greater psychological well-being. Generally, physical activity helps reduce stress hormone levels [35] and contributes to an improved emotional state. However, a systematic literature review indicates that evidence supporting the impact of increased physical activity and reduced sedentary behavior on psychological well-being among youth remains limited [36].

In this study, MVPA was also analyzed alongside sports participation, which showed a positive correlation with greater cardiorespiratory fitness. Supporting this, research on older adults (mean age 50) found that sports-related physical activity is linked to a slower decline in cardiorespiratory fitness over ten years [37]. Most sports commonly played by school students—such as basketball, football, tennis, and padel—are aerobic in nature and thus improve the specific fitness components they train. Additionally, a review study reported that sports participation not only offers benefits like stronger bones, reduced body fat, lower risk of cardiovascular disease, and increased muscular endurance and strength but is also associated with higher levels of cardiorespiratory fitness [38].

Physical inactivity is one of the five leading causes of mortality [39]. It is related to the development of various chronic diseases [40]. It may predict these conditions both independently of and alongside physical activity. In the current study, which involved young school students with an average age of about 18 years, no significant associations were found between physical inactivity and physical health indicators such as cardiorespiratory fitness or body mass index. This is likely because the onset of chronic diseases develops gradually over time, which a cross-sectional design cannot capture.

However, the study did reveal clear associations between higher levels of physical inactivity and poorer mental health outcomes, including increased psychological distress and reduced psychological well-being. The relationship between physical inactivity and mental health may be bidirectional. Some research suggests that poor mental health leads to greater physical inactivity, which then contributes to deteriorating physical health across samples aged 15 and older [41]. Other studies indicate that sedentary time directly predicts depressive symptoms and indirectly affects them via irregular sleep patterns [42]. Consistent with these findings, another study confirmed that mental health status is worse among physically inactive individuals compared to those who are physically active [43]. Additionally, sedentary behavior combined with low levels of vigorous physical activity has been linked to depression, anxiety symptoms, and dissatisfaction with school life among Chinese youth [44]. These findings support the applicability of physical activity interventions as effective strategies for reducing depressive symptoms and improving mental health [45].

Approximately 29 percent of contemporary Lithuanian potential conscripts report experiencing high psychological distress. This figure has tripled over the past five years compared to pre-pandemic levels of psychological distress among conscripts in 2018 [3]. Additionally, one in five potential conscripts currently experiences poor psychological well-being, and nearly six percent are at risk for depression. The mental health of young people has worsened since the COVID-19 pandemic. In 2016, only one in five Lithuanian high school students reported high psychological distress [46]. Although rates increased during the COVID-19 lockdown and decreased afterward, they have not returned to pre-pandemic levels.

Meanwhile, cardiorespiratory fitness among school students in Lithuania has steadily declined over the past 30 years since monitoring began [3,47], with a particularly sharp decrease of as much as 29 percent observed in the 18-year-old group (unpublished data; project report, 2024). Alongside declining physical fitness levels, physical activity among adolescents has also slightly decreased, which may explain the reduction in cardiorespiratory fitness. Although physical activity levels have recently stabilized, reaching a plateau, only 23 percent of high school students meet the WHO guidelines for children and adolescents, which recommend at least 60 min of moderate-to-vigorous physical activity daily [5]. Applying the adult PA guidelines (starting at age 18), which require at least 150 min of moderate-to-vigorous physical activity per week, the current study found that only 31 percent of 17 to 19-year-old school students meet this threshold. In comparison, 55 percent of conscripts of similar age were sufficiently active before military service in 2018 [3]. In contrast, current school students’ nutritional status has improved since 2016 (36 percent versus 14 percent, respectively) [48]. This improvement may be attributed to multiple policy changes in Lithuanian schools since 2016 that promote healthy nutrition. These comparisons suggest that while nutrition policies have effectively supported healthier diets, significant work remains to enhance physical activity among youth.

Implementing measures such as providing only healthy food options by eliminating unhealthy ones, redesigning school environments to encourage physical activity, offering various physically active options during school breaks, and increasing the number of weekly physical education classes could further promote positive behavioral changes.

This study also found that cardiorespiratory fitness and BMI are interconnected, with both being significantly associated with psychological distress. Specifically, individuals with lower cardiorespiratory fitness and higher BMI tend to experience greater levels of psychological distress. These findings are consistent with those reported in previous studies [29,49]. Altogether, these findings highlight the close interrelationship between physical and mental health, emphasizing that both aspects are critical for overall well-being. In the context of military service, maintaining optimal physical fitness and a healthy BMI is especially important, as both physical and psychological health play vital roles in ensuring effective performance and resilience among service members.

This study contributes valuable insights to the fields of public health, military medicine, and exercise science. It expands our understanding of how behavioral and health-related factors influence the physical and mental well-being of young adults eligible for conscription. These insights can inform the development of evidence-based strategies and interventions aimed at improving health and fitness outcomes in this population, ultimately contributing to more effective recruitment, training, and retention processes within military settings. Moreover, the findings have broader implications for public health initiatives targeting youth, emphasizing the importance of holistic approaches that integrate physical activity, mental health support, and healthy lifestyle promotion. This study also highlights the need for ongoing research to track the long-term effects of such interventions, ensuring sustained benefits throughout the critical transition from adolescence to adulthood.

### Limitations

The cross-sectional design of this study does not permit the determination of causal relationships. Nonetheless, the findings remain important, especially considering the need to improve both health behaviors and overall health among potential conscripts. Physical activity and nutrition were measured through self-reports, which may have introduced some inaccuracies. However, the large sample size helps mitigate this limitation, as individual reporting errors tend to balance out, resulting in more reliable overall estimates. Due to the time-consuming nature of the study for both the children and their teachers-who had to allocate time within their teaching schedules for the research-some potentially significant confounding factors may have remained unmeasured. As a result, no covariates were included in the regression models. However, the findings from this homogeneous sample, consisting solely of young males of similar age, can still be considered robust and reliable as they reflect the current state of the art in this specific population.

## 5. Conclusions

The physical and mental health of the potential conscripts is interrelated, and both are related to health-enhancing behaviors. Implementing behavior change interventions and policies in school settings could significantly benefit potential conscripts and youth more broadly by promoting improvements in both physical and mental health. Engaging multiple stakeholders, including families, teachers, and community health professionals, in behavior change interventions can enhance their effectiveness and sustainability. Training school staff and involving external experts can improve the quality and impact of health promotion programs. Regular monitoring of health indicators—such as physical fitness and body mass index—at least once per year, combined with brief mental health screening and assessment of behavioral factors, could help identify early signs of deterioration and enable timely preventive measures. This type of monitoring system, including both genders, could be integrated into an electronic diary platform, allowing parents and students to access individual results while providing summarized data to school administrators, regional policymakers, and researchers for informed decision-making and targeted interventions.

Future longitudinal research is essential to evaluate the effects of such behavioral policies and practices over time, thereby providing evidence to confirm the causal relationships between health behaviors and health outcomes.

## Figures and Tables

**Table 1:** Descriptive statistics of study variables.

Study Variables	Percent	Median (IQR)
Cardiorespiratory fitness		6.00 (3.00)
Risk zone	22.0	
Needs improvement zone	43.5	
Good fitness zone	34.5	
Body mass index		22.34 (3.48)
Underweight	7.2	
Normal	73.7	
Overweight	13.1	
Obesity	6.0	
Psychological distress		17.00 (7.00)
High	28.7	
Low	71.3	
Psychological well-being		60.00 (24.00)
Risk of depression	5.6	
Poor	20.9	
Good	73.4	
Nutrition		7 (4.00)
Poor	24.1	
Average	40.0	
Good	35.9	
MVPA		2.00 (1.00)
Not sufficient	73.5	
Sufficient	26.5	
Sports		
Does not engage	34.8	
Engages	65.2	

Note: MVPA—moderate-to-vigorous physical activity.

**Table 2:** The relationship between physical and mental health and health behaviors.

Health Behaviors	Physical Health		Mental Health	
	Cardiorespiratory Fitness	Body Mass Index (Overweight or Obese)	Psychological Distress	Psychological Well-Being
	Std. Slope	Odds Ratio	Std. Slope	Std. Slope
Nutrition	0.133 [0.03–0.14] *	1.09 [1.03–1.16] ***	−0.061 [−0.18–0.02]	0.172 [0.14–0.34] **
MVPA	−0.049 [−0.34–0.12]	0.98 [0.91–1.10]	0.058 [−0.14–0.64]	0.150 [0.30–1.10] **
Sports (engages)	0.202 [0.10–0.30] ***	0.67 [0.44–1.02]	0.077 [−0.02–0.32]	0.026 [−0.12–0.23]
Time spent passively	0.045 [−0.03–0.11]	1.05 [−0.99–1.12]	0.145 [0.12–0.34] **	−0.131 [−0.34–−0.11] ***
R^2^; *p*	0.054; <0.001	0.033; <0.05	0.036; >0.05	0.11; <0.001

Note: MVPA—moderate-to-vigorous physical activity; * *p* < 0.05; ** *p* < 0.01; *** *p* < 0.0001.

**Table 3:** Correlations among physical and mental health indicators.

	Cardiorespiratory Fitness	Body Mass Index	Psychological Distress
Cardiorespiratory fitness	1		
Body mass index	−0.314 **	1	
Psychological distress	−0.104 *	0.141 **	1
Psychological well-being	0.054	0.038	0.374 **

Note: * *p* < 0.05; ** *p* < 0.01.

## Data Availability

The data presented in this study are available on request from the corresponding author due to privacy.
